# Evaluation of hepatoprotective activity of *Cissus quadrangularis* stem extract against isoniazid-induced liver damage in rats

**DOI:** 10.4103/0253-7613.71920

**Published:** 2010-12

**Authors:** A.H.M. Viswanatha Swamy, Rucha V. Kulkarni, A.H.M. Thippeswamy, B.C. Koti, Aparna Gore

**Affiliations:** Department of Pharmacology, K.L.E. University’s College of Pharmacy, Vidyanagar, Hubli 580 031, Karnataka, India

**Keywords:** Antioxidant activity, *Cissus quadrangularis*, hepatotoxicity, isoniazid

## Abstract

**Objective:**

The study was designed to investigate the hepatoprotective activity of methanol extract of *Cissus quadrangularis* (CQ) against isoniazid-induced hepatotoxicity in rats.

**Materials and Methods:**

The successive petroleum ether (60–80°C) and methanol extracts of *C. quadrangularis* were used. Hepatic damage was induced in Wistar rats by administering isoniazid (54 mg/kg, p.o.) once daily for 30 days. Simultaneously, CQ (500 mg/kg p.o) was administered 1 h prior to the administration of isoniazid (54 mg/kg, p.o.) once daily for 30 days. Silymarin (50 mg/kg p.o) was used as a reference drug.

**Results:**

Elevated levels of aspartate transaminase, alanine transaminase, alkaline posphatase, and bilirubin following isoniazid administration were significantly lowered due to pretreatment with CQ. Isoniazid administration significantly increased lipid peroxidation (LPO) and decreased antioxidant activities such as reduced glutathione, superoxide dismutase, and catalase. Pretreatment of rats with CQ significantly decreased LPO and increased the antioxidant activities.

**Conclusion:**

The results of this study indicated that the hepatoprotective effect of CQ might be attributed to its antioxidant property.

## Introduction

Tuberculosis is a communicable disease and spread easily among people. Over one-third of the world’s population is infected with *Mycobacterium tuberculosis*, and over 2 million people per year will die of the disease.[1] A meta-analysis of studies involving several anti-tuberculosis drug regimens estimates the incidence of liver toxicity is 2–6% with co-administered isoniazid and rifampicin and 1.6% with isoniazid alone.[2] The administration of isoniazid produces many metabolic and morphological aberrations in liver. Isoniazid can cause mild-to-moderate elevation of serum transaminases in approximately 10–20% of patients and severe hepatotoxicity in approximately 0.5–2%.[3] The isoniazid-induced hepaotoxicity is initiated by CYP-450-mediated metabolism to toxic metabolites such as acetylhydrazine and hydrazine. Hydrazine reacts with sulfhydryl group, which results in glutathione (GSH) depletion within the hepatocytes leading to cell death. These bioactive metabolites are produced by a series of enzymes and induction of oxidative stress. CYP2E1 is involved in isoniazid-induced hepatotoxicity in human and animals by generation of free radicals.[4,5]

*Cissus quadrangularis* Linn. (Family: Vitaceae) is an edible plant, commonly known as “bone setter” found in hotter parts of India, Sri Lanka, East Africa, Malaysia, and Thailand. The stout quadrangular stem is traditionally used for treatment of bone fracture, piles, chronic ulcers, asthma, scurvy, irregular menstruation, constipation, and blindness.[6] Phytochemical review shows the presence of two tetracyclic triterpenoids, β-sitosterol, δ-amyrin, isopentacosanoic acid, flavonoids such as quercetin, kaempferol, steroidal principles, β-carotene, and vitamin C.[7] The plant has been extensively studied for its antimicrobial activity,[8] neuropharmacological effects,[9] gastroprotective effect,[10] anti-nociceptive activity,[11] and bone healing.[12]

Therefore, this study has been designed to evaluate the hepatoprotective activity of methanol extract of *C. quadrangularis* stem (CQ) against isoniazid-induced liver damage in albino rats.

## Materials and Methods

### Animals

Wistar albino rats of either sex weighing 150–200 g were procured from animal house of K.L.E.S.’s College of Pharmacy, Hubli, were used for the study after the approval of Institutional Animal Ethics Committee. They were housed in clean polypropylene cages under standard conditions of temperature 25 ± 2 °C and 12 h light/12 h dark cycle and fed with standard diet (Gold Mohur Lipton India Ltd.) and water *ad libitum*.

### Plant material

The stem of *Cissus quadrangularis* was collected from the local area of Dharwad and Hubli of Karnataka, and was authenticated at the Department of Botany of H.S. Kothambri Science Institute, Hubli and a voucher specimen has been deposited at the herbarium for further reference (SKA.HSK/Auth/227/2009-10).

### Processing of plant material

Above material was shade dried at room temperature and was subjected to size reduction to get coarse powder. The powdered material was subjected to successive extraction in a Soxhlet apparatus using solvents petroleum ether (60–80 °C) and methanol. Appearance of colorless solvent in the siphon tube was taken as the end-point of extraction. The extracts were concentrated to ¾ of its original volume by distillation. The yield was 10.8% w/w and 14.8% w/w for petroleum ether and methanol extract, respectively. Methanol extract of *Cissus quadrangularis* stem was evaluated for hepatoprotective and antioxidant activity. The hepatotoxicity was induced by isoniazid. The parameters monitored in this study are aspartate amino transferase (AST), alanine aminotransferase (ALT) and alkaline phosphate (ALP), total bilirubin (TB), and direct bilirubin in serum. Malano di aldehyde (MDA), glutathione (GSH), superoxide dismutase (SOD), and catalase (CAT) in liver were also monitored.

All the drugs, chemicals, and reagents were procured from S.D Fine Chemicals, Mumbai, India. Pure silymarin was kindly provided by Micro Labs, Bangalore, India. All the chemicals were of analytical grade. Kits for biochemical estimation were obtained from ERBA Diagnostics, Daman, India.

Animals were divided into four groups containing six animals in each. Group I (control) rats were fed with standard diet and were administered with an aqueous solution of 1% CMC (1 mL/kg p.o.) once daily for a period of 30 days. In Group II (isoniazid control), rats were treated with isoniazid (54 mg/kg p.o.) once daily for a period of 30 days. Group III rats were treated with methanol extract of CQ at (500 mg/kg p.o.) and receive isoniazid (54 mg/kg p.o.) 1 h after administration of CQ extract once daily for a period of 30 days. Group IV rats were treated with standard drug silymarine (50 mg/kg p.o) and receive isoniazid (54 mg/kg p.o) 1 h after administration of standard drug once daily for a period of 30 days. At the end of the experiment, rats were sacrificed and blood was collected for the separation of serum.

### Assessment of liver damage

Blood was collected under ether anesthesia 24 h after last dose and analyzed for serum AST, ALT, ALP, TB and direct bilirubin using ERBA diagnostics kits. The liver tissue was dissected out immediately, washed with ice cold saline and used for the determination of lipid peroxidation (LPO),[13] GSH,[14] SOD[15] and CAT.[16]

### Histopathological investigations

The liver tissues were dissected out and fixed in 10% formalin. The paraffin sections were prepared and stained with hematoxylin and eosin and examined using light microscopy.

### Statistical analysis

The results were expressed as the mean ± SEM. The results were analyzed using one-way ANOVA followed by Dunnett’s multiple comparison tests. Data were computed for statistical analysis using Graph Pad Prism Software. Differences between the data were considered significant at *P* < 0.05.

## Results

Isoniazid-treated animals showed a marked increase in the level of marker enzymes AST, ALT, ALP, and bilirubin total and direct [Table 1]. The increase was statistically significant as compared with control group. Pretreatment of rats with methanol extract of CQ reduced the level of marker enzymes and bilirubin significantly. The antioxidant liver enzyme levels in isoniazid treatment group showed a significant increase in LPO, while a significant decrease in GSH, SOD, and CAT when compared to control group as shown in Table 2. Pretreatment with methanol extract of CQ significantly prevented the increase in LPO (*P* < 0.001) and decrease in GSH (*P* < 0.05), SOD (*P* < 0.05), and CAT (*P* < 0.001).

**Table 1:** Effect of methanol extract of Cissus quadrangularis on biochemical parameters in isoniazid-induced hepatotoxicity

*Treatment*	*AST, IU/L*	*ALT, IU/L*	*ALP, IU/L*	*Total bilirubin, mg/dL*	*Direct bilirubin, mg/dL*
Control	53.63 ± 3.06	45.22 ± 2.20	133.2 ± 3.60	0.78 ± 0.04	0.25 ± 0.03
Isoniazid control (54 mg/kg)	93.71 ± 3.37^b^	134.3 ± 6.04^b^	250.8 ± 7.93^b^	0.94 ± 0.03^a^	0.44 ± 0.03^b^
Isoniazid + methanol extract of CQ (54 mg/kg + 500 mg/kg)	63.52 ± 3.10***	95.81 ± 0.02***	203.2 ± 10.11*	0.82 ± 0.02*	0.30 ± 0.01***
Isoniazid + silymarin (54 mg/kg + 50 mg/kg)	60.24 ± 2.53***	87.73 ± 2.5***	153.9 ±; 0.35***	0.79 ± 0.02**	0.28 ± 0.01***

Data were analyzed by ANOVA followed by Dunnett’s test. Each value is the mean ± SEM; *n* = 6,

a P < 0.01,

b P < 0.001 when compared with control group.

* P < 0.05

** P < 0.01

*** P < 0.001 when compared with isoniazid group.

**Table 2:** Antioxidant activity of *Cissus quadangularis* stem extract in isoniazid-induced hepatotoxicity in rats

*Treatment*	*MDA (nmoles/mg protein)*	*GSH (nmoles/mg protein)*	*Superoxide dismutase activity (B/mg protein)*	*Catalase activity (A/mg protein)*
Control	2.27 ± 0.22	6.97 ± 1.06	12.76 ± 1.36	71.96 ± 2.13
Isoniazid control (54 mg/kg)	4.67 ± 0.35^a^	3.67 ± 0.35^a^	6.36 ± 1.08^a^	46.68 ± 2.18^a^
Isoniazid + methanol extract of CQ (54 mg/kg + 500 mg/kg)	3.08 ± 0.18***	6.47 ± 0.66*	10.68 ± 0.99*	60.64 ± 2.64***
Isoniazid + silymarin (54 mg/kg + 50 mg/kg)	2.78 ± 0.18***	6.83 ± 0.66*	10.74 ± 1.08*	67.13 ± 1.59***

Data were analysed by ANOVA followed by Dunnett’s test. Each value is the mean ± SEM; *n* = 6,

a P < 0.001 when compared to control group.

* P < 0.05,

*** P < 0.001 when compared with isoniazid group. A: μ mole of H_2_O_2_ consumed/minute. B: One unit of activity was taken as the enzyme reaction, which gave 50% inhibition of NBT reduction in 1 min.

Histological profile of control animals showed normal architecture with central veins and portal triad. Animals treated with isoniazid exhibited inflammation, centrilobular necrosis, and sinus congestion. Pretreatment with methanol extract of CQ reduced the inflammation, centrilobular necrosis, and sinus congestion induced by isoniazid.

## Discussion

Isoniazid is metabolized into the bioactive metabolites hydrazine and acetylisoniazid followed by hydrolysis to acetylhydrazin which is oxidized into hepatotoxic intermediaries by CYP 450. ALT, AST, and ALP are well known diagnostic indicators of hepatic injury.[17] Increased levels of these enzymes in serum of the isoniazid-treated animals indicate liver damage as these enzymes leak out from liver into blood due to tissue damage. Pretreatment with methanol extract of CQ, the levels of these marker enzymes in serum were near normal, which may be a consequence of the stabilization of plasma membrane as well as repair of hepatic tissue damage caused by isoniazid. In liver injury due to hepatotoxin, there is a defective excretion of bile by the liver which is reflected in their increased levels in serum.[18] Hepatotoxicity is characterized by cirrhotic liver condition which in turn increased the bilirubin release,[19] which was observed in isoniazid treatment group (Group II). Pretreatment with CQ restored the level of bilirubin to near normal status, suggesting the possibility that the extract stabilises biliary dysfunction of rat liver, which is a clear indication of the improvement of the functions of the liver cells.

There is evidence that antitubercular drugs cause cellular damage through the induction of oxidative stress, a consequence of dysfunction of hepatic antioxidant defense system.[20] The depletion of antioxidant defenses and/or rise in free radical production deteriorates the prooxidant–antioxidant balance, leading to oxidative stress-induced cell death. MDA was one of the main LPO products, its elevated levels could reflect the degrees of LPO injury in hepatocytes.[21] The increase in MDA level in isoniazid-treated rats indicates enhanced peroxidation leading to a failure of the antioxidant defense mechanism to prevent formation of excess free radicals.[22] Pretreatment with CQ prevented significantly LPO either directly or through GSH by scavenging the free radicals.

In oxidative stress, GSH is converted into glutathione disulfide (GSSG) and depleted leading to LPO. Therefore, the role of GSH is as a marker for the evaluation of oxidative stress. Decreased GSH levels in isoniazid administered rats may be due to its increased utilization. Pretreatment with methanol extract of CQ restored the GSH levels. It may be understood that the effect of CQ may be due to an initial reduction in hepatic peroxidative activities, thereby leading to restoration of the GSH content. It is known that SOD and CAT constitute a mutually supportive team of antioxidant enzymes, which provides a defense system against ROS. In this study, SOD decreased significantly in isoniazid-treated animals due to an excessive formation of superoxide anions. The activity of H_2_O_2_ scavenging enzyme CAT is also decreased significantly after isoniazid treatment. The decline in these enzyme levels can be explained by the fact that excessive superoxide anions may inactivate SOD, thus, resulting in an inactivation of the H_2_O_2_ scavenging enzyme. Pretreatment with methanol extract of CQ effectively prevented the decrease in SOD and CAT activities, which may be attributed to the scavenging of radicals by CQ resulting in protection of these enzymes.

Histopathological observation shows CQ has reduced focal hemorrhage, inflammation, centrilobular degeneration, and necrosis. The treatment with CQ normalized the isoniazid-induced biochemical and histopatholgical changes, therefore it is suggested that hepatoprotective activity of methanol extract of CQ against isoniazid challenge might be due to its property of reducing oxidative stress. This may be due to its various antioxidant constituents such as quercetin and kaempferol, β-carotene, and vitamin C.

## Conclusion

CQ possesses hepatoprotective activity against isoniazid-induced liver damage. The major mechanisms may be responsible for the hepatoprotective activity of CQ are restoration of tissue GSH, SOD, and CAT levels, indicating that the in-built protective mechanism is being restored which may be due to the presence of phytochemical constituents in CQ.
